# Polymicrobial infections: Do bacteria behave differently depending on their neighbours?

**DOI:** 10.1080/21505594.2018.1426520

**Published:** 2018-05-04

**Authors:** Iñigo Lasa, Cristina Solano

**Affiliations:** Laboratory of Microbial Pathogenesis, Navarrabiomed, Universidad Pública de Navarra (UPNA), Complejo Hospitalario de Navarra (CHN), IdiSNA, Irunlarrea 3. Pamplona, Navarra, Spain

**Keywords:** Staphylococcus aureus, exoproteome, polymicrobial infections, microbial community, competition

## Abstract

Despite the number of examples that correlate interspecies interactions in polymicrobial infections with variations in pathogenicity and antibiotic susceptibility of individual organisms, antibiotic therapies are selected to target the most relevant pathogen, with no consideration of the consequences that the presence of other bacterial species may have in the pathogenicity and response to antimicrobial agents.

In this issue of Virulence, Garcia-Perez et al. [10] applied replica plating of used wound dressings to assess the topography of distinct *S. aureus* types in chronic wounds of patients with the genetic blistering disease epidermolysis bullosa, which is characterized by the development of chronic wounds upon simple mechanical trauma. This approach led to the identification of two strains of *S. aureus* coexisting with *Bacillus thuringiensis* and *Klebsiella oxytoca. S. aureus* is highly prevalent in chronic wound infections, whereas *B. thuringiensis* and *K. oxytoca* are regarded as opportunistic pathogens. These bacterial species did not inhibit each other's growth under laboratory conditions, suggesting that they do not compete through the production of inhibitory compounds. Using a top-down proteomic approach to explore the inherent relationships between these co-existing bacteria, the exoproteomes of the staphylococcal isolates in monoculture and co-culture with *B. thuringiensis* or *K. oxytoca* were characterized by Mass Spectrometry.

Bacteria living on internal and external surfaces of the human body are typically surrounded by different strains and species with whom they compete for scarce nutrients and limited space. The exception to this rule comes only at very specific places, such as the gastric epithelia, which as far as we know is colonized only by *Helicobacter pylori*, or when bacteria traverse the epithelia and colonize internal organs to cause infection. In the former cases, bacteria grow as single species and competition only occurs between siblings. Although competition for resources between individuals of the same species is frequent as evidenced by the rapid emergence of mutants with advantageous traits for growing in a particular niche [1], the intrinsic clonality of single species populations make them insensitive to more complex interference competition mechanisms. Thus, complex competitive phenotypes are usually adopted by members of one species that, either alone or working cooperatively, develop strategies to outcompete and displace members of other species [2]. The two major ways of interspecies competition are on one hand, exploitative competition, which implies one species consuming a limiting resource and thus, restricting the growth of the competitor, and on the other, interference competition, where one species produces antimicrobial compounds that damage neighbouring cells.

Genomic analysis confirms that competition is highly prevalent in bacteria because most bacterial genomes include a significant number of genes for the production of secondary metabolites and other potentially damaging molecules, that include antibiotics, molecules that inhibit quorum sensing, surfactants, exopolysaccharides, proteases and type VI secretion systems (for review see [3]). For example, it has been shown that *Pseudomonas aeruginosa* secreted factors can alter *S. aureus* susceptibility to different antibiotics [9]. The presence of a *P. aeruginosa* population producing LasA endopeptidase or rhamnolipids makes *S. aureus* more susceptible to vancomycin and tobramycin, respectively. Because the production of these molecules by *P. aeruginosa* is highly strain dependent, the behavior of *S. aureus* during the treatment of polymicrobial infections will vary depending on the genotype of the coexisting *P. aeruginosa* strain in the infected tissue.

However, growing in polymicrobial communities does not always implies competition and bacterial species sharing the same niche can also promote each other's growth [4]. Positive interactions, termed mutualism or syntrophy, have been related with exchange of metabolites, in which one partner provides a metabolite that is consumed by the other in exchange for a reward [5]. A particular case of mutualistic microbial interaction involves the protection of one microbe by another in the context of antibiotic resistance [6,7]. The mechanisms by which some sensitive bacteria are protected against antibiotics by neighboring microbes include the formation of protective structures, namely biofilms, and the secretion of compounds that deactivate the antibiotic. For example, the presence of the fungal pathogen *Candida albicans* or its secreted cell wall polysaccharide material induces *S. aureus* biofilm formation and increases the bacterium's tolerance to antibiotic killing [8].

Despite the number of examples that correlate interspecies interactions in polymicrobial infections with variations in pathogenicity and antibiotic susceptibility of individual organisms, antibiotic therapies are selected to target the most relevant pathogen, with no consideration of the consequences that the presence of other bacterial species may have in the pathogenicity and response to antimicrobial agents.

In this issue of Virulence, Garcia-Perez et al [10]. applied replica plating of used wound dressings to assess the topography of distinct *S. aureus* types in chronic wounds of patients with the genetic blistering disease epidermolysis bullosa, which is characterized by the development of chronic wounds upon simple mechanical trauma. This approach led to the identification of two strains of *S. aureus* coexisting with *Bacillus thuringiensis* and *Klebsiella oxytoca. S. aureus* is highly prevalent in chronic wound infections, whereas *B. thuringiensis* and *K. oxytoca* are regarded as opportunistic pathogens. These bacterial species did not inhibit each other's growth under laboratory conditions, suggesting that they do not compete through the production of inhibitory compounds. Using a top-down proteomic approach to explore the inherent relationships between these co-existing bacteria, the exoproteomes of the staphylococcal isolates in monoculture and co-culture with *B. thuringiensis* or *K. oxytoca* were characterized by Mass Spectrometry.

The results revealed that *S. aureus* exoproteomes contain a significantly lower amount of exoproteins upon co-culturing with *K. oxytoca* or *B. thuringiensis.* Interestingly, this decrease was particularly evident in the case of extracellular proteins with a predicted cytoplasmic localization. The reduction in extracellular proteins during co-culturing might be due to an enhanced proteolysis, to the consumption of these proteins by the other organism in respective co-cultures or to a decrease in *S. aureus* cell lysis. As regards proteolysis, it is not easy to explain why this process would preferentially degrade cytoplasmic proteins, amongst all extracellular proteins. On the other hand, and from an evolutionary point of view, lysis of a percentage of the population within a monospecies population would represent a beneficial trait or ‘public good’ for the community, whereas the benefits of the same process in a multi-species community are certainly questionable. In this respect, it would be interesting to determine how often *S. aureus* coexists in the same space with *K. oxytoca* and *B. thuringiensis*, since cooperation is more common between species that have a shared evolutionary history and less common between those with no recent interactions [11].

A question that immediately arises from the finding that the content of *S. aureus* extracellular proteins is dramatically altered when this bacterium is grown in the presence of other bacterial species is how recognition of other bacterial species is carried out and also, which is the signal transduction system responsible for connecting the presence of other bacterial species with a change in bacterial physiology. In this respect, it would be interesting to use the proteomic approach of this pioneering study to compare the extracellular proteomes of a collection of *S. aureus* mutants deficient in two-component systems grown either in monoculture or in the presence of other bacterial species.

Even though evidence of competition or cooperation between bacteria that colonize and invade the human body and the existing microbiota and other pathogens is growing, our understanding about how relationships between different bacterial species occur still remains preliminary. Therefore, additional studies are needed to understand how the findings of this manuscript can be generalized and which is their biological relevance. Needless to say, two co-isolated strains that are found to compete in the laboratory may actually live separated by millimeters in a wound and therefore, sampling may exaggerate competition relationships between strains. Thus, it would be very important to develop sampling methods that conserve spatial structure (for instance, MALDI Imaging mass spectrometry), where the identity and protein profile of individual groups of cells over different areas and over time could be followed.

## References

[1] Stewart PS, Franklin MJ Physiological heterogeneity in biofilms. Nat Rev Micro. 2008;6:199–210. doi:10.1038/nrmicro1838 18264116

[2] Hibbing ME, Fuqua C, Parsek MR, et al. Bacterial competition: Surviving and thriving in the microbial jungle. Nat Rev Micro. 2010;8:15–25. doi:10.1038/nrmicro2259 PMC2879262 19946288

[3] Ghoul M, Mitri S The Ecology and Evolution of Microbial Competition. Trends in Microbiology. 2016;24:833–45. doi:10.1016/j.tim.2016.06.011 27546832

[4] Friedman J, Gore J Ecological systems biology: The dynamics of interacting populations. Current Opinion in Systems Biology. 2017;1:114–21. doi:10.1016/j.coisb.2016.12.001

[5] Morris BEL, Henneberger R, Huber H, et al. Microbial syntrophy: interaction for the common good. FEMS Microbiology Reviews. 2013;37:384–406. doi:10.1111/1574-6976.12019 23480449

[6] Yurtsev EA, Conwill A, Gore J Oscillatory dynamics in a bacterial cross-protection mutualism. Proc Natl Acad Sci U S A. 2016;113:6236–41. doi:10.1073/pnas.1523317113 27194723 PMC4896713

[7] Perlin MH, Clark DR, McKenzie C, et al. Protection of *Salmonella* by ampicillin-resistant *Escherichia coli* in the presence of otherwise lethal drug concentrations. Proceedings of the Royal Society B: Biological Sciences. 2009;276:3759–68. doi:10.1098/rspb.2009.0997 19656787 PMC2817278

[8] Kong EF, Tsui C, Kucharíková S, et al. Commensal Protection of *Staphylococcus* *aureus* against Antimicrobials by *Candida albicans* Biofilm Matrix. mBio. 2016;7:e01365–16. doi:10.1128/mBio.01365-16 27729510 PMC5061872

[9] Radlinski L, Rowe SE, Kartchner LB, et al. *Pseudomonas aeruginosa* exoproducts determine antibiotic efficacy against *Staphylococcus aureus*. PLoS Biol. 2017;15:e2003981. doi:10.1371/journal.pbio.2003981 29176757 PMC5720819

[10] García-Pérez AN, de Jong A, Junker S, et al. From the wound to the bench: exoproteome interplay between wound-colonizing *Staphylococcus aureus* strains and co-existing bacteria. Virulence. 2018;9:363–378. doi: 10.1080/21505594.2017.1395129. PMC5955179 29233035

[11] Tan CH, Lee KWK, Burmølle M, et al. All together now: experimental multispecies biofilm model systems. Environ Microbiol. 2017;19:42–53. doi:10.1111/1462-2920.13594 27878947

